# Sex Differences in Affective Dysfunction and Alterations in Parvalbumin in Rodent Models of Early Life Adversity

**DOI:** 10.3389/fnbeh.2021.741454

**Published:** 2021-11-04

**Authors:** Seneca N. Ellis, Jennifer A. Honeycutt

**Affiliations:** ^1^Program in Neuroscience, Bowdoin College, Brunswick, ME, United States; ^2^Department of Psychology, Bowdoin College, Brunswick, ME, United States

**Keywords:** early life adversity, parvalbumin, sex differences, estrogens, testosterone, anxiety, depression, development

## Abstract

The early life environment markedly influences brain and behavioral development, with adverse experiences associated with increased risk of anxiety and depressive phenotypes, particularly in females. Indeed, early life adversity (ELA) in humans (i.e., caregiver deprivation, maltreatment) and rodents (i.e., maternal separation, resource scarcity) is associated with sex-specific emergence of anxious and depressive behaviors. Although these disorders show clear sex differences in humans, little attention has been paid toward evaluating sex as a biological variable in models of affective dysfunction; however, recent rodent work suggests sex-specific effects. Two widely used rodent models of ELA approximate caregiver deprivation (i.e., maternal separation) and resource scarcity (i.e., limited bedding). While these approaches model aspects of ELA experienced in humans, they span different portions of the pre-weaning developmental period and may therefore differentially contribute to underlying mechanistic risk. This is borne out in the literature, where evidence suggests differences in trajectories of behavior depending on the type of ELA and/or sex; however, the neural underpinning of these differences is not well understood. Because anxiety and depression are thought to involve dysregulation in the balance of excitatory and inhibitory signaling in ELA-vulnerable brain regions (e.g., prefrontal cortex, amygdala, hippocampus), outcomes are likely driven by alterations in local and/or circuit-specific inhibitory activity. The most abundant GABAergic subtypes in the brain, accounting for approximately 40% of inhibitory neurons, contain the calcium-binding protein Parvalbumin (PV). As PV-expressing neurons have perisomatic and proximal dendritic targets on pyramidal neurons, they are well-positioned to regulate excitatory/inhibitory balance. Recent evidence suggests that PV outcomes following ELA are sex, age, and region-specific and may be influenced by the type and timing of ELA. Here, we suggest the possibility of a combined role of PV and sex hormones driving differences in behavioral outcomes associated with affective dysfunction following ELA. This review evaluates the literature across models of ELA to characterize neural (PV) and behavioral (anxiety- and depressive-like) outcomes as a function of sex and age. Additionally, we detail a putative mechanistic role of PV on ELA-related outcomes and discuss evidence suggesting hormone influences on PV expression/function which may help to explain sex differences in ELA outcomes.

## Introduction

Adversity in early life is widespread (Finkelhor et al., 2013) and places individuals at an increased risk for developing later-life psychiatric disorders, such as anxiety and depression (Gatt et al., 2009; Nugent et al., 2011; Heim and Binder, 2012). Further, experiencing adverse environmental stressors during early development and/or childhood has been linked to impaired cognitive function and maladaptive behavioral outcomes (Chapman et al., 2004; Krugers et al., 2017; Vaiserman and Koliada, 2017), with onset often occurring in a protracted manner, years after the adverse experience (Spertus et al., 2003; Hagan et al., 2015; Russell et al., 2018). Early-life adversity (ELA) manifests in a variety of instances and includes both physical and sexual abuse, emotional/psychological abuse, adverse family circumstances, neglect, poverty, and other environmental factors (Felitti et al., 1998; Kuhlman et al., 2020). The 2021 report released by the National Child Abuse and Neglect Data System (NCANDS) found that approximately 4.4 million children in the United States received a Child Protective Services referral of suspected abuse or neglect in 2019, with over 650,000 identified victims of abuse/neglect (U.S. Department of Health and Human Services, Administration for Children and Families, Administration on Children, Youth and Families, Children’s Bureau, 2021). It is important to note that these numbers are likely lower than the actual number of abuse or neglect cases, as most instances of child abuse or neglect go unreported. Early life experiences play a significant role in shaping short- and long-term outcomes regarding both cognitive-behavioral and neural development (Kundakovic and Champagne, 2015; Chen and Baram, 2016). While it is clear that a history of ELA is a significant risk factor in the development of affective disorders (Hoppen and Chalder, 2018), the underlying mechanism(s) by which ELA confers this risk remain largely unknown. Therefore, it is critical that we leverage findings from preclinical models to identify putative neurobiological drivers of sex-specific risk following ELA to reveal windows of opportunity for individualized intervention and/or treatment.

Several rodent models of ELA exist to approximate distinct aspects and types of adverse experiences to elucidate the role of adversity on neural and behavioral consequences across the lifespan. One widely used model leverages maternal separation (MS) as an analog of early caregiver deprivation during the postnatal and pre-weaning periods. MS protocols typically involve the removal and isolation of pups from dam and littermates for a designated period of time over a series of days, typically ranging from 3–4 h per day from postnatal day (P) 2 to 20 (e.g., Grassi-Oliveira et al., 2016; Coley et al., 2019; Köhler et al., 2019; Honeycutt et al., 2020; Drastichova et al., 2021), though some research groups only maintain separations for the first 14 days of life (e.g., Uchida et al., 2010; Callaghan and Richardson, 2011; Teissier et al., 2020). This ELA model is widely used as it closely models early life caregiver deprivation seen in institutionalized rearing (Kundakovic and Champagne, 2015), and MS in rodents also shows outcomes comparable to those in humans with a history of abuse (Teicher et al., 2006; Nemeroff, 2016). Importantly, this model approximates early life psychosocial neglect which is one of the most prevalent forms of ELA in the United States, accounting for approximately 78% of mistreatment cases (National Scientific Council on the Developing Child, 2012). Another widely utilized model of ELA is the limited bedding (LB) paradigm, which aims to model resource scarcity (Molet et al., 2014). Increasing evidence suggests that the LB model results in disruption of maternal behavior, thereby leading to fragmented, abuse-like, and unpredictable maternal care (Ivy et al., 2008; Rice et al., 2008; Walker et al., 2017). While these are both models of ELA, it is clear that the *type* of adversity model used (and therefore the specific type of adversity experienced) impacts both neural and behavioral outcomes (Murthy and Gould, 2018; Brenhouse and Bath, 2019; Demaestri et al., 2020).

There is undeniable evidence suggesting that biological sex plays an important—and alarmingly understudied—role in both short- and long-term outcomes following adversity in both humans (e.g., Altemus et al., 2014; Colich et al., 2017; LoPilato et al., 2019) and rodent models (e.g., Bath, 2020; Eck et al., 2020; Honeycutt et al., 2020). In humans, women are more likely than men to develop anxiety-related disorders in their lifetime (Kessler et al., 1994), with anxiety in women more likely to be clinically significant (McLean et al., 2011). Because ELA is associated with an increased risk of anxiety-related outcomes in both humans and rodent models, it is important that we understand the disparate sex-specific outcomes to better approach individualized risk assessment and treatment. Preclinical findings suggest that male mice with a history of MS show no changes in social interaction following ELA, while MS females show increased social interaction and increased anxiety-like behaviors (Bondar et al., 2018). Interestingly, in this same study MS males exhibited significant variability in locomotor activity, which may account for some of the effects observed. Despite clear evidence for sex differences in affective disorders, most studies examining affect-and, in fact, most studies across behavioral neuroscience-have looked only at males, neglecting to include females or to explicitly consider sex as a biological variable (SABV; Shansky, 2019). As such, more research is needed to understand sex differences following ELA to: (1) better model mental illness in preclinical assays; (2) address glaring sex differences in symptom onset and patient outcomes; and (3) determine neurobiological drivers of affective dysfunction in an attempt to identify putative targets for intervention and treatment. In this review, we shed light on sex differences as observed in preclinical ELA models (specifically, MS and LB models) and discuss their possible interactions with identified neural markers of pathological risk and circuit dysfunction.

There are several neural changes thought to contribute to deleterious behavioral outcomes associated with anxiety and depression: two affective disorders that show increased risk of emergence following exposure to ELA (Nugent et al., 2011; Pagliaccio and Barch, 2016). One widely observed neural change involves alterations in overall inhibitory/GABAergic function (Page and Coutellier, 2019; Prévot and Sibille, 2021), which are likely exacerbated by adverse experiences (Maguire, 2014). Parvalbumin (PV), a calcium-binding protein expressed within a specific subset of GABAergic neurons, is thought to be involved in affective dysregulation characteristic of anxiety (e.g., Page et al., 2019; Xiao et al., 2020) and depression (e.g., Perova et al., 2015; Thaweethee-Sukjai et al., 2019). Indeed, reduced PV levels are associated with increased anxiety-like behavior and affective dysfunction in rodent studies (Godavarthi et al., 2014; Lussier and Stevens, 2016; Xu et al., 2016; Todorović et al., 2019; Vojtechova et al., 2021) and indirectly in human studies looking at Tourette syndrome, which is thought to be closely related to anxiety (Kalanithi et al., 2005; Kataoka et al., 2009). A multitude of studies have also found a reduction in PV interneurons in the hippocampus (HPC), prefrontal cortex (PFC), and basolateral amygdala (BLA), all of which are thought to be important for affective regulation following ELA (Leussis et al., 2012; Wieck et al., 2013; Ganguly et al., 2015; Grassi-Oliveira et al., 2016; Gildawie et al., 2021).

The goal of the present review is to synthesize the limited amount of prior work examining PV outcomes at the intersection of ELA and sex, to identify patterns that might explain how these factors contribute to affective outcomes. Specifically, we address disparate observations in PV outcomes following ELA that might be mediated by sex hormones, adversity type, and/or the timing of adversity/tissue collection. A discussion on the developmental time course of PV outcomes alongside changes in sex hormone and receptor levels is also presented to evaluate a possible relationship that may help to explain the observed sex-specific effects of ELA.

### Parvalbumin

PV is a calcium-binding protein that supports the fast-spiking phenotype of PV-expressing neurons, a property that ideally positions them for synchronizing the activity of surrounding cells (Sohal et al., 2009; Chen et al., 2017; Kawaguchi et al., 2019). PV-containing neurons are the most abundant subtype of GABAergic interneurons in the central nervous system, accounting for ~40% of all neocortical GABAergic neurons (Rudy et al., 2011). These PV cells are characterized as fast-spiking with low input resistance, leading to a rapid sequence of action potentials (Kawaguchi and Kubota, 1997; Woodruff and Sah, 2007). The high frequencies of action potentials, in addition to their perisomatic synapses on target cells, allow for the synchronization of electrical activity by orchestrating the timing of principal neuron spiking (Freund and Buzsáki, 1996; Woodruff and Sah, 2007). This synchronization plays an important role in the excitatory/inhibitory (E/I) tone of individual neurons as well as regional activity, which is thought to be altered by ELA (Singh-Taylor et al., 2015; Ohta et al., 2020). There are two distinct subtypes of PV-expressing cells: basket cells, which target proximal dendrites and their soma, and chandelier cells, which target synapses on the axon initial segment (Kawaguchi and Kubota, 1997). Both subtypes significantly contribute overall E/I tone in target neurons/regions (Ferguson and Gao, 2018), and therefore are well-positioned to orchestrate neuronal ensembles of activity. There is mounting evidence suggesting that ELA in rodent models leads to a decrease in PV cells in various regions of the brain, particularly the PFC (Brenhouse and Andersen, 2011; Leussis et al., 2012; Wieck et al., 2013; Holland et al., 2014; Ganguly et al., 2015; do Prado et al., 2016; Grassi-Oliveira et al., 2016), the HPC (Murthy et al., 2019), and the BLA (Gildawie et al., 2020). Given the orchestrating role of PV cells, these alterations in PV expression and/or function may contribute to some of the aberrant cognitive and neurobehavioral outcomes of ELA associated with neuronal inhibition and affective dysfunction, as seen in depression, schizophrenia, and anxiety (Brown et al., 2015; Gonzalez-Burgos et al., 2015; Zou et al., 2016; Perez et al., 2019; Murthy and Gould, 2020). However, it is noteworthy to underscore the variability in PV outcomes following ELA that are likely mediated by methodological differences in ELA application (i.e., MS vs. LB), age of tissue collection, species, and sex. We have provided an overview of PV outcomes in Table 1 that details these findings with an emphasis on implemented methodology and PV levels, as well as related behavioral outcomes.

**Table 1:** PV and behavioral outcomes as a function of sex, age, and ELA type.

							PV Outcome		Anxiety and Depressive Behaviors	
Study	Sex	Species	Type of ELA	Age of ELA	Age of Brain Collection	PV Meas.	Male	Female	Male	Female
**Short-Term Maternal Separation (1–12 days) and/or Early Weaning**										
Murthy et al. (2019)	M	Mouse	MS +Early weaning (P17)	P2–16	P60-P70	IHC	↓HPC (ventral)	-	↑ anxiety (EPM) ↑ activity (NE)	-
Katahira et al. (2018)	M	Mouse	MS: 1 day, 24 hs	P4	P4, P5, P14, P28	IHC	↓(left HPC on P14 and P28)	-	-	-
Aksic et al. (2021)	M	Rat	MS: 1 day, 24 h	P9	P60	IHC	↓(CA1, PFC)	-	-	-
Giachino et al. (2007)	M	Rat	MS: 12 days 3 h/day	P2–14	P35	IHC	↑(LA) n.c. (HPC, BLA)	-	↓ (SI)	-
Richardson et al. (2021)	M + F	Rat	MS: 12 days 3 h/day	P2–14	P18	IHC	n.c. (PFC)	n.c. (PFC)	-	-
**Long-Term Maternal Separation (18–19 days)**										
Soares et al. (2020)	M + F	Rat	MS: 18 days 4 h/day	P2–20	P20	IHC	n.c. (PFC, CA1, DG) ↓(BLA, CA3)	n.c. (PFC, CA1, DG) ↓(BLA, CA3)	-	-
Gildawie et al. (2020)	M + F	Rat	MS: 18 days 4 h/day	P2–20	P20, P40, P70	IHC	↓(BLA at P40) n.c. (PFC)	n.c. (PFC, BLA)	-	-
Gildawie et al. (2021)	M + F	Rat	MS: 18 days 4 h/day	P2–20	P85	IHC	n.c. (PFC)	n.c. (PFC)	n.c. (EZM)	n.c. (EZM)
Brenhouse and Andersen (2011)	M	Rat	MS: 18 days 4 h/day	P2–20	P25, P40	WB, IHC	↓(PFC at P40)	-	↓Working memory (W/S)	-
Lukkes et al. (2017)	F	Rat	MS: 18 days 4 h/day	P2–20	P41	WB	-	↓(PFC, BLA, DR)	-	↑ depression (LH)
Lukkes et al. (2018)	F	Rat	MS: 18 days 4 h/day	P2–20	P41	WB	-	↓(Amygdala, PFC)	-	n.c. (LH)
Wieck et al. (2013)	M	Rat	MS: 18 days 4 h/day	P2–20	P40	WB, IHC	↓(PFC)	-	-	-
Ganguly et al. (2015)	M	Rat	MS: 18 days 4 h/day	P2–20	P43	IHC	↓(PFC)	-	↑ anxiety (EPM, OFT)	-
Leussis et al. (2012)	M + F	Rat	MS: 18 days 4 h/day	P2–20	P40, P100	WB, IHC	↓(PFC at P40)	↓(PFC at P40)	↑ depression (LH)	↑ depression (LH)
Holland et al. (2014)	M + F	Rat	MS: 18 days 4 h/day	P2–20	P25–27 or P42–45	WB	↓ (PFC in adolescence)	↓ (PFC in juvenility)	↓ (SI in adolescence)	↓ (SI in juvenility)
do Prado et al. (2016)	M + F	Rat	MS: 18 days 4 h/day	P2–20	P56	WB	↓(PFC)	n.c. (PFC)	↓Working memory (W/S)	-
Grassi-Oliveira et al. (2016)	M + F	Rat	MS: 18 days 4 h/day	P2–20	P40	IHC	↓(PFC)	n.c. (PFC)	↓Working memory (W/S)	↓Working memory (W/S)
(Kim et al. 2020)	M	Rat	MS: 19 days 3 h/day	P2–21	Adolescent	WB, IHC	WB: n.c. (HPC) IHC: ↓(HPC)	-	↓ anxiety (OFT) ↑ depression (FST)	-
**Limited Bedding Model (7–10 days)**										
Manzano-Nieves et al. (2020)	M	Mouse	LB: 7 days	P4–11	P16, 21, 28, 50, 75	IHC	↑(BLA at P21; PFC at 75)	-	n.c. (EZM)	n.c. (EZM)
Bath et al. (2016)	M	Mouse	LB: 7 days	P4–11	P16, P21, P28	IHC	↑(PFC)	-	Accelerated contextual fear suppression	-
Goodwill et al. (2018)	M + F	Mouse	LB: 7 days	P4–11	P8, P12, P16, P21	IHC	n.c. (OFC) n.c. (PFC)	↓(OFC); n.c. (PFC)	n.c. Cognitive Function (S/S)	↓ Cognitive Function (S/S)
Guadagno et al. (2020)	M + F	Rat	LB: 10 days	P1–10	P28–29	IHC	n.c. (BLA)	n.c. (BLA)		

*The included studies are all those that have explicitly examined PV outcomes following ELA in brain regions associated with affective dysfunction. Here, we report each study and identify the sex of subjects (male (M) or female (F); species (Rat or Mouse); type of ELA (maternal separation (MS) or limited bedding (LB) and adversity postnatal (P) day timeframe); the age of brain tissue collection; brain regions examined [basolateral amygdala (BLA), hippocampus (HPC) and HPC subfields where specified (CA1, CA3, DG), lateral amygdala (LA), orbitofrontal cortex (OFC), and prefrontal cortex (PFC)]; and method of PV quantification [western blot (WB) or immunohistochemistry (IHC)]. PV outcomes are divided based on subject sex, with no change (n.c.) between ELA and controls indicated if a lack of significant effect was observed. Directionality of behavioral outcomes related to PV changes are also detailed based on sex, as well as assay used [elevated zero maze (EZM), forced swim test (FST), novel environment (NE), social interaction (SI), set shifting (S/S), and win/shift (W/S)]. Directionality of behavioral and neural effects are represented as a decrease (↓), increase (↑), or no change (n.c.) compared to controls*.

In rodent models, ELA generally leads to a decrease in PV-expression in the PFC in rats (e.g., Brenhouse and Andersen, 2011; Leussis et al., 2012; Wieck et al., 2013; Holland et al., 2014; Ganguly et al., 2015; do Prado et al., 2016; Grassi-Oliveira et al., 2016; Lukkes et al., 2017), the orbitofrontal cortex in mice (Goodwill et al., 2018), the HPC in both rats and mice (e.g., Katahira et al., 2018; Murthy et al., 2019), and the BLA in rats (e.g., Lukkes et al., 2017, 2018; Gildawie et al., 2020), all of which are regions considered to be key mediators of anxiety- and depressive-like behaviors (Kent and Rauch, 2003; Bannerman et al., 2004; Bertoglio et al., 2006; Pandya et al., 2012; Huang et al., 2018). These decreases in PV cells in the HPC (e.g., Murthy et al., 2019) have also been associated with increases in anxiety-like behaviors within the elevated plus maze (EPM) in male mice. The work outlined in Table 1 constitutes all relevant research, to our knowledge, that has looked at PV outcomes following ELA (specifically, MS or LB). Of note, out of all studies looking at ELA induced effects on PV (*n* = 22), only 10 included both males and females in their analyses (with an additional two studies looking only at female subjects), underscoring the need for ELA studies to use SABV in methodological approaches to understand how sex mediates adversity-related outcomes.

**Figure 1:**
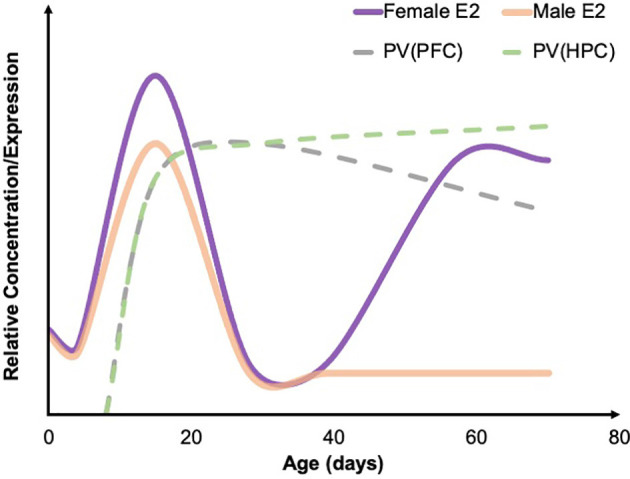
Developmental trajectories of estradiol and PV. A summary of the developmental trajectory of estradiol (E2) concentrations in male (solid orange) and female (solid purple) rodents across early postnatal development through young adulthood, as well as the general developmental trajectory of PV levels in the PFC (dashed gray) and HPC (dashed green). Both males and females see a spike in E2 around P15. PV concentrations across development have not been well studied; however, existing developmental work suggests that PV levels in the HPC steadily increase through early adulthood, while PV levels remain relatively stable in the PFC. Figure adapted from data presented in Bell (2018), de Lecea et al. (1995), and Du et al. (2018). PFC, prefrontal cortex; HPC, hippocampus; PV, parvalbumin.

Indeed, in some studies, ELA leads to marked differences in PV expression that are sex-specific, and it is important to note that PV expression and/or staining intensity reportedly varies by sex across brain regions including the PFC, BLA, and HPC (e.g., Blurton-Jones and Tuszynski, 2002; Wu et al., 2014; Soares et al., 2020), with some reports showing similar developmental trajectories in control rats (e.g., Gildawie et al., 2020). In typical mice, there are some documented differences in the developmental trajectory of PV between males and females. In both the dorsal HPC and the ventral HPC females see a continuous increase in PV from week 3 to week 12 of age. This consistent increase is not observed in male mice, whose PV levels appear to remain constant from week 3 to week 12 after an initial pre-weaning surge (Wu et al., 2014; Ueda et al., 2015). However, sex differences in PV development have not been particularly well characterized; see Figure 1 for a generalized normative trajectory of PV protein expression across age in typically developing rodents. In relation to ELA, male rats are more likely to have a decrease in PV cells in the BLA (Gildawie et al., 2020) and the PFC (do Prado et al., 2016; Grassi-Oliveira et al., 2016). There also may be a difference in timing, as males experience a decrease in PV cells in the PFC during adolescence while females show a decrease in PV expression in the PFC during juvenility following ELA (Holland et al., 2014). Sex differences also appear in the developmental trajectory of PV (Wu et al., 2014; Du et al., 2018), which may explain some of the variability in results. Again, however, few studies have looked at the difference in PV development between males and females, particularly as it relates to ELA. Taken together, prior work suggests that PV likely plays a significant role in the outcomes associated with ELA and is, therefore, a key protein to further characterize within this context. It is possible that alterations in PV expression and/or function significantly contribute to ELA-related affective dysfunction across the lifespan and are influenced by sex hormones to drive sex-specific individual outcomes following adversity, which will be further discussed in this review.

### Adversity Type

The type of adversity impacts acute and chronic outcomes—spanning molecular to functional domains—following ELA, with evidence clearly borne out in recent work for review (see Brenhouse and Bath, 2019). Here, we focus on two widely used models of ELA: MS and LB, with MS being most closely associated with caregiver deprivation and LB being most closely associated with resource scarcity and infant maltreatment. Evidence suggests that there are differences in ELA type on later PV outcomes. Specifically, MS has been associated with a decrease in PV cells in the HPC (Katahira et al., 2018; Murthy et al., 2019), the BLA (Lukkes et al., 2017; Gildawie et al., 2020), and the PFC (Leussis et al., 2012; Wieck et al., 2013; Ganguly et al., 2015). Conversely, LB has been associated with an increase in PV in the BLA (Manzano-Nieves et al., 2020), PFC (Manzano-Nieves et al., 2020), and HPC (Bath et al., 2016). However, decreased PV following LB has been observed in the OFC of females but not males (Goodwill et al., 2018). The mechanisms underlying these opposing findings in response to different models of ELA remain unknown but may be influenced by length and timing of experience. Typically, LB models last for 7 days, while MS models last for 18 days, and it is, therefore, possible that the difference in duration of adversity contributes to the disparity observed between adversity types. MS stress may lend itself more to isolation stress, while LB may lead to more aversive stress, as modified LB methods have been related to abusive maternal care (e.g., Lewin et al., 2019). It is possible that the types of stress elicited by the models differ, leading to unique changes in PV.

Sex may also be a factor to consider when evaluating the impact of adversity type. One study looking at sex differences following ELA using the LB model found that cognitive ability, *via* rule shifting, was impaired more in females than in males (Goodwill et al., 2018). This same study also revealed that female rats have a decrease in PV levels in the OFC following LB, while male rats see no change. This is a different pattern of results than seen in studies that use an MS model, which often find that males experience a long-term decrease in PV following adversity while females do not (e.g., Holland et al., 2014; do Prado et al., 2016; Grassi-Oliveira et al., 2016). This suggests that the type of adversity may also affect males and females differently. However, it is worth noting that there are few studies that have directly compared males and females in this way, and therefore this sex disparity may be biased due to a lack of relevant research.

While the type and timing of ELA appear to markedly influence later life outcomes when presented during the pre-weaning period, the length of the experience during that time may also be important. Prolonged ELA experience, as modeled by MS up until weaning, confers increased risk of PV decreases and/or dysfunction, with most of the past research reporting decreased PV levels following ELA *via* MS (see Table 1). This general decrease in PV expression after ELA is also associated with concomitant changes in anxiety- and depressive-like behaviors (e.g., Leussis et al., 2012; Grassi-Oliveira et al., 2016), suggesting a possible link between these outcomes.

### Developmental Age

An important factor that warrants consideration when evaluating PV expression following ELA is the age at which the animal experiences ELA, as well as the age of tissue collection and evaluation. In rodents experiencing adversity in early development (P0–20) or even juvenility (P20–35) there were generally decreases in PV levels in the PFC and HPC, particularly after MS (see Table 1). However, this was true for when brain tissue was collected in adolescence (approx. P35–50) or young adult/adulthood (approx. P50-P70) following ELA, but not during juvenility (Holland et al., 2014; do Prado et al., 2016; Grassi-Oliveira et al., 2016). This indicates that both the timing of adversity and the age of brain collection are important factors to consider. Adult rodents, however, were found to have increased PV levels following chronic stress in adulthood in the PFC (Shepard et al., 2016; Shepard and Coutellier, 2017), suggesting that age may have differing effects on the brain’s response to stress. It is possible that stress occurring after the brain has fully developed elicits a different response than stress that occurs while the brain is still undergoing major development (Romeo and McEwen, 2007). PV cell density increases in the PFC throughout juvenility and adolescence before decreasing again in adulthood (Ueno et al., 2017). Therefore, stress that occurs during juvenility or adolescence may impact the development of PV cells, while stress in adulthood may have a compensatory effect and leads to an increase in PV cells.

There is also evidence suggesting that ELA, specifically MS, may have a delayed impact on PV cell density. Brains that were collected immediately (or within a few days) after ELA experience generally had no significant differences in PV levels compared to control-rearing (Giachino et al., 2007; Brenhouse and Andersen, 2011; Soares et al., 2020; Richardson et al., 2021), while brains collected later in life (in adolescence and/or P40 and older) generally had decreased PV levels (Leussis et al., 2012; Wieck et al., 2013; Ganguly et al., 2015; Lukkes et al., 2017, 2018; Kim et al., 2020; Aksic et al., 2021). Since PV cells have been found to increase significantly in number from juvenility to adolescence in brain regions such as the PFC (Caballero et al., 2014; Wu et al., 2014), it is possible that ELA may inhibit the maturational time course of PV cells. However, this regulation may also be regionally dependent, as some groups have identified decreases in HPC PV across development from juvenility to adolescence in typical rats (e.g., Honeycutt et al., 2016), making the dynamic nature of PV protein expression even more apparent. Furthermore, developmental timing of other insults (for instance, exposure to NMDA antagonists which appear to disproportionately impact PV cell functionality; (Kinney et al., 2006; Abekawa et al., 2007), also mediate PV outcomes in an age-dependent manner (e.g., Honeycutt and Chrobak, 2018). This research suggests that there may be a compounding effect of ELA and downstream neural changes that lead to the decrease in PV levels following adversity. Furthermore, as males and females have slightly different developmental timelines, it is important to consider sex as a variable when looking at developmental age and ELA effects.

### Sex Hormones

An important area of consideration, which has more recently been gaining traction in the field, for ongoing and future research is to systematically investigate sex differences in relation to ELA. The majority of ELA research (and admittedly, research in general) has focused on the investigation of male subjects. However, males and females have different physiological and behavioral responses to ELA (Donner and Lowry, 2013; Maeng and Milad, 2015; Perry et al., 2021), as previously mentioned. For example, juvenile male rats did significantly worse on non-spatial and spatial memory tasks following MS, while female rats had no impairment (Frankola et al., 2010). Social and aggression-related behavior following ELA also differs between males and females (Farrell et al., 2016). Prior work in rodents has shed some light on these differences, suggesting that MS in mice leads to opposite effects in adult offspring, with MS males showing decreased latency to attack a resident intruder, and lactating MS females showing decreased latency in the same task (Veenema et al., 2007), with ELA also leading to increased aggression/play-fighting in male rats (Veenema and Neumann, 2009). Therefore, it is important to consider how sex hormones may be interacting with underlying putative mechanisms, including PV cells, to identify possible relationships that may drive sex-specific risk and/or resilience following ELA.

#### Estrogens and Aromatase

Estrogens may be a potential explanation for the sex differences observed in PV levels following ELA. Estrogens play many important roles in brain function, including the modulation of neurotransmitters (Herbison, 1997; Rubinow et al., 1998), cognition (Sherwin and Henry, 2008; Albert and Newhouse, 2019), and synaptic plasticity (Albert and Newhouse, 2019). Estrogen receptors (ER) are crucial in the development and behavioral outcomes in both males and females (e.g., Hess and Cooke, 2018), including behaviors such as fear extinction and aggression (Ogawa et al., 1997; Scordalakes and Rissman, 2003; Graham and Milad, 2014). Estrogens have been implicated in contributing to anxiety-related outcomes (Borrow and Handa, 2017) and depression (Albert and Newhouse, 2019), with decreased levels of estrogens in the PFC (Shansky et al., 2004), amygdala (Walf and Frye, 2006), and HPC (Xu et al., 2015, 2016) associated with the development of anxiety- and depressive-like behaviors following stress.

Low levels of estrogens and ERs are associated with increased anxiety behavior, both in rodents (e.g., Walf and Frye, 2006; Borrow and Handa, 2017) and in humans (e.g., Wittchen and Hoyer, 2001; Almeida et al., 2005; Solomon and Herman, 2009; Holsen et al., 2011). Estradiol and ERs, specifically ERα and ERβ, have been found to have protective effects against anxiety- (Lund et al., 2005; Walf and Frye, 2006, 2010; Filova et al., 2015) and depressive-like (Galea et al., 2001; Walf et al., 2009; Österlund, 2010) behaviors. These protective effects may be due to the role of estradiol and ERs in preventing cell death by promoting the release of anti-inflammatory proteins (Behl et al., 1995; Patrone et al., 1999; Simpkins and Dykens, 2008; Smith et al., 2011) and promoting axonal sprouting by increasing axodendritic synapse formation (Matsumoto and Arai, 1979; Kadish and Van Groen, 2002). Estradiol may also play a role in neuronal regeneration by stimulating the release of glial apolipoprotein and enhancing antioxidant mechanisms (Sudo et al., 1997; Stein, 2001; Struble et al., 2007). Finally, estradiol and ERs may also increase synaptic transmission (Garcia-Segura et al., 2001), perhaps due to the antioxidant properties of estradiol molecules (Behl et al., 1997) and the neuroprotective signal cascades that begin with binding to ERs (Sohrabji et al., 1994; Lagrange et al., 1997) or interactions with estrogens (Sohrabji et al., 1995; Singer et al., 1999; Singh et al., 1999).

This has led to the use of estrogens in several human studies as a successful treatment for anxiety and depression in women, with perimenopausal women receiving treatments of estrogens experiencing significantly lower levels of anxiety and depression (Schmidt et al., 2000; de Novaes Soares et al., 2001; Grigoriadis and Kennedy, 2002; Ancelin et al., 2007; Misra et al., 2013). While not a common treatment for anxiety and depression, the results of these studies suggest that estrogens might have potential as a successful treatment, and therefore warrants further investigation to understand the mechanism(s) by which it mediates affective function. In line with these human findings, ovariectomized female rodents show significantly more anxiety- and depressive-like behaviors, but these effects are significantly reduced following replacement of estrogens (Estrada-Camarena et al., 2003; Shansky et al., 2004; Walf et al., 2008, 2009; Walf and Frye, 2010; Kiss et al., 2012; Daendee et al., 2013; Furuta et al., 2013; Tian et al., 2013; Xu et al., 2015). Therefore, the use of estrogens, especially in women with low baseline levels of estrogens or older women who have experienced a decrease in estrogens following menopause, may be important in the treatment of anxiety and depression.

Furthermore, increasing evidence suggests that both artificial and natural increases in estrogens and ERs lead to increases in PV levels (Ross and Porter, 2002; Wu et al., 2014; Bunratsami et al., 2015). Specifically, ovariectomized rats had a decreased number of ERα and ERβ receptors as well as a decrease in PV levels. This effect was reversed following estradiol replacement (Bunratsami et al., 2015). In typical rats, the developmental trajectory of parvalbumin is very similar to the developmental trajectory of estradiol in females (Figure 1; Wu et al., 2014). There is also colocalization of ERβ and PV in inhibitory neurons located in the amygdala, HPC, cortex, and basal forebrain (Blurton-Jones and Tuszynski, 2002) and colocalization of ERα and PV in the dorsal HPC of mice (Wu et al., 2014). Therefore, a strong connection can be drawn between estrogens and PV. Additionally, the developmental time course of PV expression lines up closely with the developmental timing of estrogens fluctuations (Alcántara et al., 1993; del Río et al., 1994; de Lecea et al., 1995; Bell, 2018). In both male and female rodents, estrogens are fairly low in concentration until peaking around P15 before decreasing again and staying at relatively similar levels until females experience a pubertal increase again at P39 (Bell, 2018). In rodents, PV protein expression in most brain regions begins to emerge around P10 and typically peaks in density and intensity around P15 (Alcántara et al., 1993; del Río et al., 1994; de Lecea et al., 1995). In a large number of neocortical regions, as well as in the HPC, adult levels of PV have been observed around P21 (Alcántara et al., 1993; de Lecea et al., 1995). In females, PV levels remain consistent in the PFC throughout adolescence (Du et al., 2018). However, in males, there is a significant increase in PV in both the infralimbic and prelimbic areas of the PFC during adolescence (Du et al., 2018). One explanation for this difference is pubertal timing. Males typically begin puberty around P42, which is when an increase in PV was observed while females begin puberty earlier and experience an earlier increase in PV (Du et al., 2018). Of note, ELA *via* MS in rats has been shown to impact pubertal timing, accelerating pubertal onset in females, while delaying in males (Cowan and Richardson, 2018). However, in an LB rat model, sex hormone levels, but not pubertal onset, were impacted (Eck et al., 2020). It is therefore prudent to consider the impact of ELA-induced alterations in development as a possible driver of sex-specific neural and behavioral outcomes.

Estrogen may have specific implications for the timing of PV decreases and anxiety- and depressive-like behaviors associated with ELA. Females experience the emergence of these changes as early as juvenility, while males experience these alterations in PV expression beginning in adolescence and into adulthood (Holland et al., 2014; do Prado et al., 2016; Grassi-Oliveira et al., 2016). During puberty, females exhibit a surge in estradiol, which may explain why females exhibit altered PV levels before puberty but not after. It is possible that, in females who have experienced adversity, the estradiol surge results in a subsequent surge in PV, while also protecting against the emergence and continuation of anxiety and depression. It is important to note that males also experience a surge in estradiol during puberty; however, most of this estradiol is converted to testosterone by aromatase so the circulating levels of estradiol remain relatively low in males throughout puberty (Oyola and Handa, 2017; Bell, 2018).

While some research has looked at the role of estrogens in development, fear extinction, and aggressive behaviors in males (Ogawa et al., 1997; Scordalakes and Rissman, 2003; Graham and Milad, 2014; Hess and Cooke, 2018), few studies have considered the role of estrogens and stress in males. Interestingly, Tsuda et al. (2014) observed that ERβ knockout mice exhibit increased anxiety-like behavior, whereas male knockouts showed increased aggression that was lessened by MS. Further, MS has been associated with alterations in Erβ gene methylation in male mice (Wang et al., 2013), suggesting a role of altered estrogenic function in behavioral outcomes in both sexes. However, an important consideration is the function of aromatase, which is responsible for the conversion of testosterone to estrogens (Eck et al., 2020). This is particularly important during early-life development, as well as with the development of sexual behaviors during puberty (McCarthy, 2008; Bell, 2018). As estrogen levels are higher in males following ELA, it is possible that ELA leads aromatase to be more efficient in converting testosterone to estradiol (Eck et al., 2020), which may have a protective effect (Wei et al., 2014). Additionally, the aromatase inhibitor letrozole administered in juvenility has been associated with increased anxiety-like behavior in rats (Borbélyová et al., 2017), which suggests that decreased levels of estrogens may be an important consideration in males as well. However, more research is needed to directly determine how ELA impacts the levels and functionality of aromatase, as well as the interaction between aromatase and PV, which could be an important aspect of the sex differences that emerge after ELA.

#### Testosterone

In addition to estrogens, testosterone may also play an important preventative role in the development of anxiety and depression (Aikey et al., 2002; Buddenberg et al., 2009; Roohbakhsh et al., 2011; Giltay et al., 2012; Hodosy et al., 2012; McHenry et al., 2014). Testosterone is a hormone typically found in higher levels in males than females (Fahey et al., 1976). Males experience a surge in testosterone right before birth and maintain a moderate level of testosterone throughout juvenility until experiencing another surge during puberty (Bale and Epperson, 2017; Bell, 2018). ELA has been found to lead to a change in testosterone levels, but it is unclear whether it leads to an increase (e.g., Veenema et al., 2006; Zito et al., 2017) or decrease (e.g., Llorente et al., 2011; Tsuda et al., 2011). Interestingly, decreases in plasma testosterone were shown to be associated with less aggressive behavior in male mice, though these findings were dependent on age in 5–9 week old mice (Tsuda et al., 2011).

Testosterone has been observed to have anxiolytic and antidepressant effects in both males and females (Goldstat et al., 2003; Miller et al., 2009; Zarrouf et al., 2009; McHenry et al., 2014). Males with hypogonadism, which leads to a decrease in testosterone levels, have significantly higher rates of anxiety and depression (Shores et al., 2004; Zarrouf et al., 2009; Wainwright et al., 2011; Aydogan et al., 2012). Furthermore, hormone replacement therapy in men with a testosterone deficiency prevents or alleviates anxiety and depression (Wang et al., 1996; Seidman and Rabkin, 1998; Seidman et al., 2001; Pope et al., 2003; Kanayama et al., 2007; Zarrouf et al., 2009; Jung and Shin, 2016). Similarly, gonadectomized male rodents exhibit increased anxiety and depressive-like behavior, which is reversed following testosterone replacement treatment (Frye and Seliga, 2001; Edinger and Frye, 2005; Carrier and Kabbaj, 2012; Carrier et al., 2015). While little research has been conducted looking at testosterone therapy in women, a small number of studies found promising evidence that low-dose testosterone treatment led to a decrease in depressive-like behavior in SSRI treatment-resistant women of various ages with major depressive disorder (Miller et al., 2009) and reduced fear potentiated startle in healthy women (Hermans et al., 2006). Furthermore, low levels of salivatory testosterone may lead to an increased risk of females developing anxiety and depression (Carrier et al., 2015).

Despite the potential role of testosterone in anxiety and depression, little research has found clear interactions between PV and testosterone, which is in contrast to the overlap that is seen with PV and estrogens. One study looking at canaries found that an increase in testosterone was associated with higher levels of PV in the HVC, robust nucleus of the arcopallium, and Area X, which are all regions associated with bird song (Cornez et al., 2020). Another study in mice found no interaction between PV and testosterone (Wu et al., 2014), which may explain why males see a decrease in PV but females do not, following ELA. As previously discussed, PV colocalizes extensively with ERs, suggesting that higher availability or ERs within key brain regions may be capable of modulating PV function through ER signaling (Blurton-Jones and Tuszynski, 2002; Wu et al., 2014), whereas testosterone may not have the ability to modulate PV function this robustly. However, the anxiolytic and antidepressant roles of testosterone, specifically in promoting neuroplasticity, may explain differences in anxiety and depressive-like behavior between sexes (Carrier and Kabbaj, 2012; Wainwright et al., 2016; Walther et al., 2019), although these differences may depend on the behavioral assays used (Polanza, 2001; Scholl et al., 2019).

## Discussion

ELA is a prevalent issue globally, and its contribution to individual risk of developing later-life psychiatric disorders places an undue burden on society at large. A potential mechanism of ELA-associated outcomes (such as affective dysfunction) may be a reduction in PV expression in the PFC and HPC. However, these changes in PV levels are not ubiquitous and appear to be differentially impacted by adversity type, age, and sex. Here, we detail that the two of the most prominent models of ELA, MS and LB, have markedly different effects on PV outcomes both acutely and in the long-term. LB models have led to increases in PV in the PFC (Bath et al., 2016; Manzano-Nieves et al., 2020) of male mice, and the OFC of female, but not male rats (Goodwill et al., 2018). Conversely, MS leads to a general decrease in PV in the PFC (Leussis et al., 2012; Wieck et al., 2013; Ganguly et al., 2015) and HPC (Katahira et al., 2018; Murthy et al., 2019) of rats, which differs based on sex (see Table 1). It is likely that both the duration of the two ELA models (i.e., 7 vs. 18 days), as well as the type of adversity conferred by these models, lead to different neural outcomes.

Age of adversity also plays an important role in PV levels, with pre-weaning adversity generally leading to a decrease in PV (Holland et al., 2014; do Prado et al., 2016; Grassi-Oliveira et al., 2016), and adversity occurring in adulthood typically resulting in an increase in PV cells (e.g., Shepard et al., 2016; Shepard and Coutellier, 2017). The mechanism(s) underlying PV outcomes, as well as manifestations of anxiety- and depressive-like behaviors, as a function of both age and adversity type, remain largely unknown. Interestingly, the research overviewed here suggests males and females may be differentially susceptible to ELA-induced PV pathology, particularly following MS. Specifically, in those studies examining both sexes, female PV outcomes were either comparable to those seen in males (a decrease or no change compared to controls; Leussis et al., 2012; Guadagno et al., 2020; Soares et al., 2020; Gildawie et al., 2021; Richardson et al., 2021), or females showed a lack of PV decrease while males showed a marked decrease in PV expression following ELA (do Prado et al., 2016; Grassi-Oliveira et al., 2016; Gildawie et al., 2020). Even more intriguing, was that Holland et al. (2014) revealed an age-dependent decrease in the PFC after MS, with females showing earlier decreases in PV than males. While this disparity in findings based on sex is not yet understood, it is worth noting that PV neurons show substantial co-expression of ERs (Blurton-Jones and Tuszynski, 2002), which may serve a protective function preventing ELA-induced expression phenotypes. Indeed, administration of 17-β estradiol increased PV levels in *Pvalb* heterozygous mice (Filice et al., 2018), and estradiol administration further protects PV expression outcomes in models of ischemic brain injury (Koh, 2014). Therefore, the presence of estrogens, particularly during key points of PV development (see Figure 1), may play a protective role in neural outcomes following ELA in females. However, while this is promising, it does not account for the increased incidence of ELA-related affective dysfunction/aberrant behavior that is often observed in females, raising the possibility that alterations in PV cell function—and not simply reduced PV protein—may also be linked to ELA risk (Murthy and Gould, 2020).

In addition to underscoring the need to increase our understanding of how sex impacts ELA-associated outcomes, we detail compelling data that may suggest an overarching role of PV expression/function on ELA-related affective dysfunction. Indeed, as PV cells are well-positioned to orchestrate local circuit oscillatory patterns, it follows that significant changes in PV protein expression and/or PV neuron function would disrupt the delicate E/I balance within discrete brain regions/circuits. This careful balance of overall E/I tone is critical for mediating behavior, and therefore PV disruption leads to downstream neural and behavioral alterations characteristic of affective dysfunction (Ferguson and Gao, 2018). Taken together, we show that ELA leads to sex-dependent changes in PV outcomes, and that type/age of ELA and age of brain tissue collection further contribute to these observations. Most importantly, we also overview evidence suggesting that estrogens may serve a protective role due to colocalization of ERs on PV cells, perhaps blunting females from some of the PV-associated outcomes seen in males. Given the prevalence of ELA and the increased risk of later-life affective dysfunction, it is essential that the field recognizes that key methodological differences (i.e., age of adversity/tissue collection) and sex contribute to ELA outcomes. By systematically addressing these factors and by including SABV, we can work toward individualized prevention and treatment.

## Author Contributions

SNE and JAH both wrote the manuscript. JAH provided guidance on literature review and writing and edited the manuscript and prepared it for submission. All authors contributed to the article and approved the submitted version.

## Conflict of Interest

The authors declare that the review was conducted in the absence of any commercial or financial relationships that could be construed as a potential conflict of interest.

## Publisher’s Note

All claims expressed in this article are solely those of the authors and do not necessarily represent those of their affiliated organizations, or those of the publisher, the editors and the reviewers. Any product that may be evaluated in this article, or claim that may be made by its manufacturer, is not guaranteed or endorsed by the publisher.
